# Two HAP2-GCS1 homologs responsible for gamete interactions in the cellular slime mold with multiple mating types: Implication for common mechanisms of sexual reproduction shared by plants and protozoa and for male-female differentiation

**DOI:** 10.1016/j.ydbio.2016.05.018

**Published:** 2016-07-01

**Authors:** Marina Okamoto, Lixy Yamada, Yukie Fujisaki, Gareth Bloomfield, Kentaro Yoshida, Hidekazu Kuwayama, Hitoshi Sawada, Toshiyuki Mori, Hideko Urushihara

**Affiliations:** a Faculty of Life and Environmental Sciences, University of Tsukuba, 1-1-1 Tennodai, Tsukuba, Ibaraki 305-8572, Japan; b Sugashima Marine Biological Laboratory, Graduate School of Science, Nagoya University, 429-63 Sugashima, Toba, Mie 517-0004, Japan; c Medical Research Council Laboratory of Molecular Biology, Francis Crick Avenue, Cambridge Biomedical Campus, Cambridge CB2 0QH, UK; d Department of Biological Sciences, Graduate School of Science, The University of Tokyo, 7-3-1 Hongo, Bunkyo-ku, Tokyo 113-0033, Japan

**Keywords:** *Dictyostelium discoideum*, Macrocyst formation, Fertilization, Cell fusion protein, Sex differentiation

## Abstract

Fertilization is a central event in sexual reproduction, and understanding its molecular mechanisms has both basic and applicative biological importance. Recent studies have uncovered the molecules that mediate this process in a variety of organisms, making it intriguing to consider conservation and evolution of the mechanisms of sexual reproduction across phyla. The social amoeba *Dictyostelium discoideum* undergoes sexual maturation and forms gametes under dark and humid conditions. It exhibits three mating types, type-I, -II, and -III, for the heterothallic mating system. Based on proteome analyses of the gamete membranes, we detected expression of two homologs of the plant fertilization protein HAP2-GCS1. When their coding genes were disrupted in type-I and type-II strains, sexual potency was completely lost, whereas disruption in the type-III strain did not affect mating behavior, suggesting that the latter acts as female in complex organisms. Our results demonstrate the highly conserved function of HAP2-GCS1 in gamete interactions and suggest the presence of additional allo-recognition mechanisms in *D. discoideum* gametes.

## Highlights

• Two HAP2-GCS1 homologs are expressed in *Dictyostelium discoideum* gametes.
• Both homologs are responsible for the sexual cell fusion.
• One mating type (III) out of 3 is HAP2-GCS1-independent, corresponding to female.

## 1 Introduction

Fertilization is a central event in sexual reproduction to generate a new individual and trigger its development. Recent studies have made rapid progress in uncovering the molecules involved in this process in a variety of organisms. For example, CD9 is required on the egg (Le Naour et al., 2000, Miyado et al., 2000) and Izumo1 is required on sperm surfaces (Inoue et al., 2005) during mammalian sperm-egg fusion, and many other proteins involved in sperm and egg-coat interactions have been identified (reviewed by Ikawa et al. (2008) and Bianchi et al. (2014)). Generative lineage-specific proteins, HAP2-GCS1, are indispensable for fertilization in *Lilium* and *Arabidopsis* (Mori et al., 2006, von Besser et al., 2006), and in other organisms (reviewed by Mori et al. (2015)). In the nematode, the products of *spe* and *egg* series genes are necessary for fertilization (Singson et al., 2008). One of them, *spe45* protein, is related to Izumo1 (Nishimura et al., 2015). The similar mechanisms for allo-recognition or discrimination of self and non-self have been clarified in ascidians and self-incompatible plants (Harada and Sawada, 2008, Rea and Nasrallah, 2008, Sawada et al., 2014). Thus, it is now intriguing to consider the extent to which the mechanisms of gamete interactions are conserved across phyla.

The cellular slime mold *Dictyostelium discoideum* occupies a unique phylogenetic position, having diverged from animals after the plant lineage but before the fungal lineage (Eichinger et al., 2005). *D. discoideum* cells proliferate by fission as haploid unicellular amoebas, but gather and initiate multicellular development upon starvation to construct a fruiting body with a spore mass and a supportive stalk (Kessin, 2001). Alternatively, under dark and submerged conditions, water-sensitive spore formation is not a wise strategy for survival, and the amoebas fuse with appropriate mating-type cells to develop into dormant structures called macrocysts. Macrocyst formation is considered to be a primitive form of sexual reproduction (reviewed by Urushihara and Muramoto (2006)). There are three mating types, type-I, -II, and -III, determined by the mating-type genes, *matA, matB* and *C*, and *matS*, respectively (Bloomfield et al., 2010). To date, the molecular functions of the genes are undetermined. In addition to these genes, cell surface glycoprotein, MacA, coded by *macA* gene, is indispensable for sexual cell interactions in *D. discoideum* (Araki et al., 2012). This protein is unique to the dictyostelid lineage and its evolutionary relationship to proteins of other organisms could not be traced. Since the structure and expression of both *macA* and *matA* genes were unaltered in cell fusion-defective type-I mutants, we inferred that a third gene is involved in gamete interactions (Araki et al., 2012). A homolog of the HAP2-GCS1 gene was a candidate based on its role in gamete fusion in wide-ranging taxa.

In the present study, we detected two HAP2-GCS1 homologs in the gamete membranes of *D. discoideum* and performed their functional analysis in each mating type, and found that both of the HAP2-GCS1 homologs are indispensable for the fusion of type-I and -II gametes, but not of type-III gametes.

## 2 Materials and methods

### 2.1 Strains and cell culture

Heterothallic strains of *D. discoideum* were used. Their mating types and sexual properties are summarized in Table 1. All strains were maintained as fruiting bodies on nutrient A-medium (1/5×) agar plates with *Klebsiella aerogenes* as a food source. Axenic strains were also grown in HL5 medium containing 50 µg/ml streptomycin and either 10 µg/ml of blasticidin S (blaS) or G418 (Funakoshi, Tokyo, Japan) for the drug-resistant strains. To obtain gamete-phase cells, the growth-phase cells on an A-medium plate, which were fusion-incompetent, (IC-cells) were transferred to Bonner's salt solution (BSS) containing *K. aerogenes* and cultured on a gyratory shaker at 120 rpm in darkness at 22 °C for 16 h. These cells were referred to as fusion-competent (FC) cells.

### 2.2 Assays for sexual potency

Cellular ability with respect to macrocyst formation and cell fusion was determined following standard procedures (Urushihara, 2006). For the macrocyst assay, IC cells of two strains were mixed in a 96-well plate containing diluted *K. aerogenes* suspension in BSS and cultured in an incubator at 22 °C under darkness. Macrocysts were observed after 4–7 days. For the cell fusion assay, FC cells of the two strains were mixed in BSS at 5×10^5^ cells/ml and incubated on a gyratory shaker at 120 rpm. Following 30 min incubation, EDTA was added to a final concentration of 1 mM to stop further cell fusion, and the number of unfused cells was counted to obtain the fusion index corresponding to the percentage of cells that participated in fusion. Cell fusion indices normally exceed 60% for compatible strains.

### 2.3 Proteome analysis

Crude membrane fractions were obtained by repeated freeze-thawing of cells (Sussman and Boschwitz, 1975) and were dissolved in sample loading buffer for sodium dodecyl sulfate polyacrylamide gel electrophoresis (SDS-PAGE). For liquid chromatography-tandem mass spectrometry (LC–MS/MS) analysis, proteins were separated in a 10–20% gradient gel, size-fractionated by horizontally slicing the gel, and then digested with trypsin as described previously (Yamada et al., 2008). The digested peptides were analyzed using a capillary liquid chromatography system (Ultima3000; DIONEX, Sunnyvale, CA, USA) connected to a mass spectrometer (LTQ-XL, Thermo Scientific, Waltham, MA, USA). Raw spectrum data were processed using SEQUEST to extract peak lists, which were analyzed using the MASCOT program to query the *D. discoideum* protein database downloaded from dictyBase (Basu et al., 2013) and supplemented with MatB, MatD and MatT (*mat* locus gene products in type-II and type-III, respectively) sequences (Bloomfield et al., 2010). The sums of MASCOT scores for each protein from technical replicates were averaged and normalized among samples for a total of 1×10^5^ peptides.

### 2.4 Gene expression analysis

Total cellular RNA was extracted using RNeasy RNA Extraction Kit (Qiagen, Hilden, Germany) and converted to cDNA by PrimeScript™ II High Fidelity RT-PCR Kit (Takara Bio, Kusatsu, Japan) using random primers. PCR amplification was carried out by *KOD* Plus DNA polymerase (Toyobo, Osaka, Japan) and the primers listed in Table S1. Equality of template concentration for the PCR reaction was confirmed by amplification of *rnlA* (mitochondrial large subunit rRNA gene).

### 2.5 Transformation of *D. discoideum* cells

Transformation of axenic strains was performed by the standard electroporation procedure (Knecht and Pang, 1995) using the Transfector 800 (BTX, San Diego, CA, USA). Selection of stable transformants was carried out in HL5 containing either 10 µg/ml blaS or G418. A disruption construct was made by sequential fusion PCR (Kuwayama et al., 2002); the left and right arms were first amplified using genomic DNA as a template and the specific primer sets shown in Table S1, and the fragments were connected to the left and right of the *bsR* cassette composed of the *act*15 promoter, *bsr* gene, and *act8* terminator. An over-expression vector was constructed by inserting a cDNA between the *v18* promoter and *act8* terminator of pTMV18 (Araki et al., 2012).

### 2.6 Accessions

Accessions for individual gene and protein sequences are listed in Table S2.

## 3 Results

### 3.1 Detection of two HAP2-GCS1 homologs in the gamete membranes of *D. discoideum*

**Fig. 1:**
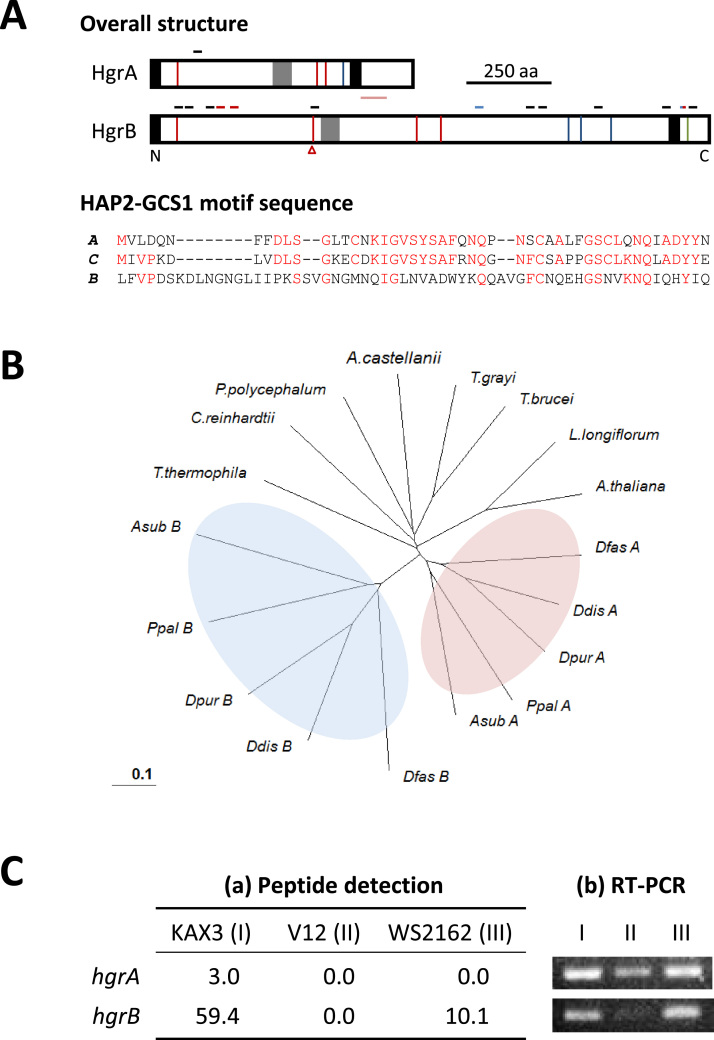
Two HAP2-GCS1 homologs in *D. discoideum*. A: The overall structures are schematically shown on top. Black boxes on the N- and near the C-termini and gray box represent a predicted signal peptide, transmembrane region, and the HAP2-GCS1 motif, respectively. Short bars in black, red, and blue above each protein indicate the approximate positions of peptides detected in KAX3, WS2162, and in both strains, respectively. Thin, perpendicular lines in green and red represent the amino acid substitutions in V12 (Type-II) and WS2162 (Type-III), respectively, and blue lines indicate common substitutions. A red line with a triangle below in HgrB indicates the position of the amino acid insertion in WS2162. A pink bar below HgrA shows the region of sequence diversity among the strains. Actual sequence variations can be seen in Figs. S1 and S2. Alignments of HAP2-GCS1 motif sequences of HgrA (*A*) (283-331) and HgrB (*B*) (496-556) to the consensus (C) are shown below. Red letter indicates match to the consensus. B: A phylogenetic tree was constructed to demonstrate weak homology between the two homologs. Pink and blue ovals represent dictyostelid orthologs of HgrA (A) and HgrB (B), respectively. HgrA sequences in other organisms were included for comparison. The accessions are listed in Table S2. Species names are shown by 4 letters for dictyostelids. C: (a) Data of HgrA and HgrB hits were extracted from the proteome analysis results of the gamete membranes (Table S3). Peptide number is normalized for 1×10^5^ total peptides in each sample. (b) Sequences for *hgrA* and *hgrB* were amplified using gamete cDNA as template and primer sets shown by dark red arrowheads in Fig. 2.

When the crude membrane fractions from FC-cells of KAX3 (type-I) were subjected to SDS-PAGE followed by LC–MS/MS analysis using *D. discoideum* protein database, we observed the expression of two HAP2-GCS1 homologs; a *D. discoideum* ortholog of HAP2-GCS1 (dictyBase ID: DDB0308506) and a weakly homologous protein, DDB0308507. We reconfirmed the gene sequences and named the two proteins HgrA (HAP2-GCS1 related protein A) and HgrB for convenience. Gene names are *hgrA* and *hgrB*, respectively. The latter was similar to HAP2-GCS1 only in the motif region. Both of them have a signal peptide at the N-terminus, the HAP2-GCS1 motif in the central region, and a probable transmembrane region near the C-terminus, suggesting membrane localization with a major extracellular portion and a short intracellular tail (Fig. 1A). Two genes for HAP2-GCS1 were previously referred to in *D. discoideum* (Hirai et al., 2008). However, our detailed examination revealed that they corresponded to sequences determined in the early stages of genome sequencing and gene model construction and were actually portions of *hgrA* (gene for HgrA) that cover different coding regions. While HgrA was highly homologous to HAP2-GCS1 proteins in other organisms, HgrB was limited to the dictyostelid lineage. Phylogenetic analysis of these two proteins suggested that they diverged before the initial radiation of the social amoebae and evolved independently (Fig. 1B).

HgrA-derived peptides were detected only in the FC-KAX3 (type-I) sample at a low level and undetectable in V12 (type-II) and WS2162 (type-III) (Fig. 1C). Hits for HgrB in KAX3 were much higher than HgrA over the size difference between 2 proteins. Nearly 20% of KAX3 hits were detected in WS2162 but none in V12. Expression of HgrA in IC-KAX3 cells was only slightly lower than FC-KAX3 cells and that of HgrB in IC-cells was nearly equal to FC-cells in KAX3 and WS2162 (Table S3). As to gene expression, similar levels of transcripts were detected in KAX3 and WS2162 for both *hgrA* and *hgrB*, but it was much lower in V12, *hgrB* amplification being almost undetectable. To see if structural variations affected the detection of peptides and transcripts in type-II and -III strains, we determined the genomic sequences of *hgrA* (Fig. S1) and *hgrB* in V12 and WS2162 (Fig. S2). It is likely that the HgrB level was slightly underestimated in WS2162 as one peptide out of 13 detected in KAX3 covers the region with a point mutation in WS2162, but sequence variations did not affect the PCR reaction. To summarize, expression of HAP2-GCS1 homologs is sex-dependent in *D. discoideum* but not as clearly as reported in other organisms. It was not highly elevated in the gamete-phase cells either.

### 3.2 Involvement of HAP2-GCS1 homologs in the mating of type-I strains

**Fig. 2:**
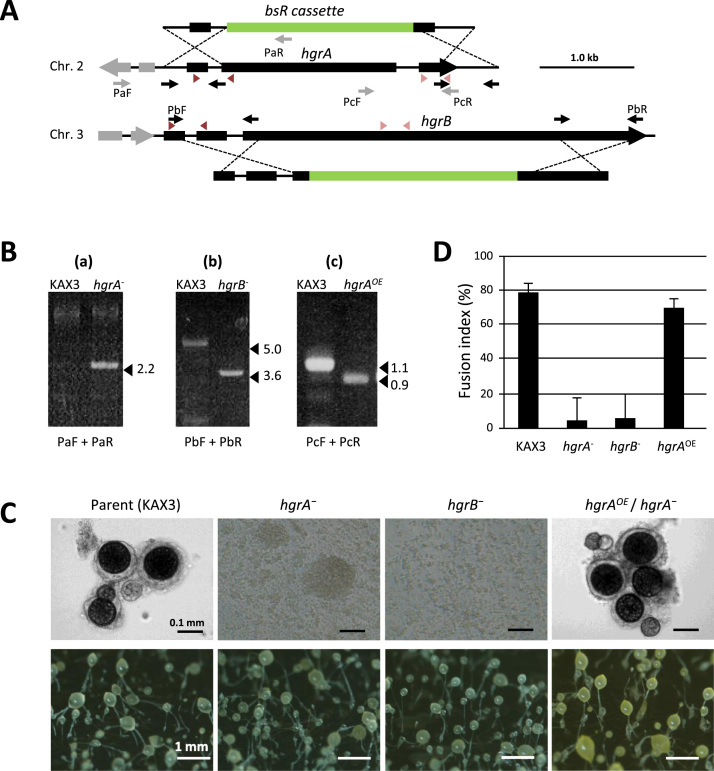
Generation of HAP2-GCS1 mutants in KAX3 and their phenotypes. A: Genome structures around *hgrA* (DDB_G0276069) and *hgrB* (DDB_G0281923) are shown together with the KO construct above or below. Gray bars on the genome represent coding sequences of the adjacent genes, and green bars, the *bsR* cassette. Thin black arrows indicate the positions of primers to amplify the left and right arms to be ligated to the bsR cassette. Thin gray arrows represent primers designed to confirm genetic modifications and dark and light red arrowheads, for RT-PCR. B: Disruption of *hgrA* (a) and *hgrB* (b) and introduction of the *hgrA* coding sequence into the *hgrA*-null mutant (c) were confirmed by PCR using the primer sets shown below each electrophoretogram. Source of template genomic DNA is shown above each lane. C: Macrocyst assay results are shown. Growth phase cells (IC-cells) for each strain were cultured with V12 cells in a 96-well plate to test macrocyst forming ability as described in the Materials and Methods (up) or plated alone with *K. aerogenes* on an agar plate (bottom). Pictures were taken after 4 days. D: Gamete-phase cells (FC-cells) of each strain were mixed with FC-cells of V12 and incubated for 30 min on a gyratory shaker to obtain fusion indices. Bars represent standard deviations of 3 measurements.

To determine if the two HAP2-GCS1 homologs are involved in *D. discoideum* mating, we disrupted *hgrA* and *hgrB* in KAX3 by homologous recombination (Fig. 2A and B). Multiple independent knockout mutants obtained for each gene showed the same phenotypes; they lacked macrocyst formation ability with type-II tester (V12), but exhibited normal growth and fruiting body formation (Fig. 2C). Since macrocyst formation includes multiple events after gamete fusion, we examined whether the mutants were defective in the process of sexual cell fusion or after that. As shown in Fig. 2D, cell fusion indices of the mutants were reduced to the background level, indicating that *hgrA* and *hgrB* were required for sexual cell fusion. As the disruption of *hgrA* or *hgrB* alone completely abolished the mating ability of KAX3, the two genes do not complement each other. The mutants were also unable to mate with type-III tester (WS2162) (data not shown). If *hgrA* cDNA was introduced to one of its null mutants, and expressed under the control of the V18 promoter, both cell fusion and macrocyst formation abilities were restored (Fig. 2B–D). Because no intermediate stages are known for *D. discoideum* gamete fusion such as formation of specialized conjugation structures, it was not possible to determine how sexual cell fusion was interrupted in the disruptants.

### 3.3 Mating-type-specific function of *hgrA* and *hgrB*

**Fig. 3:**
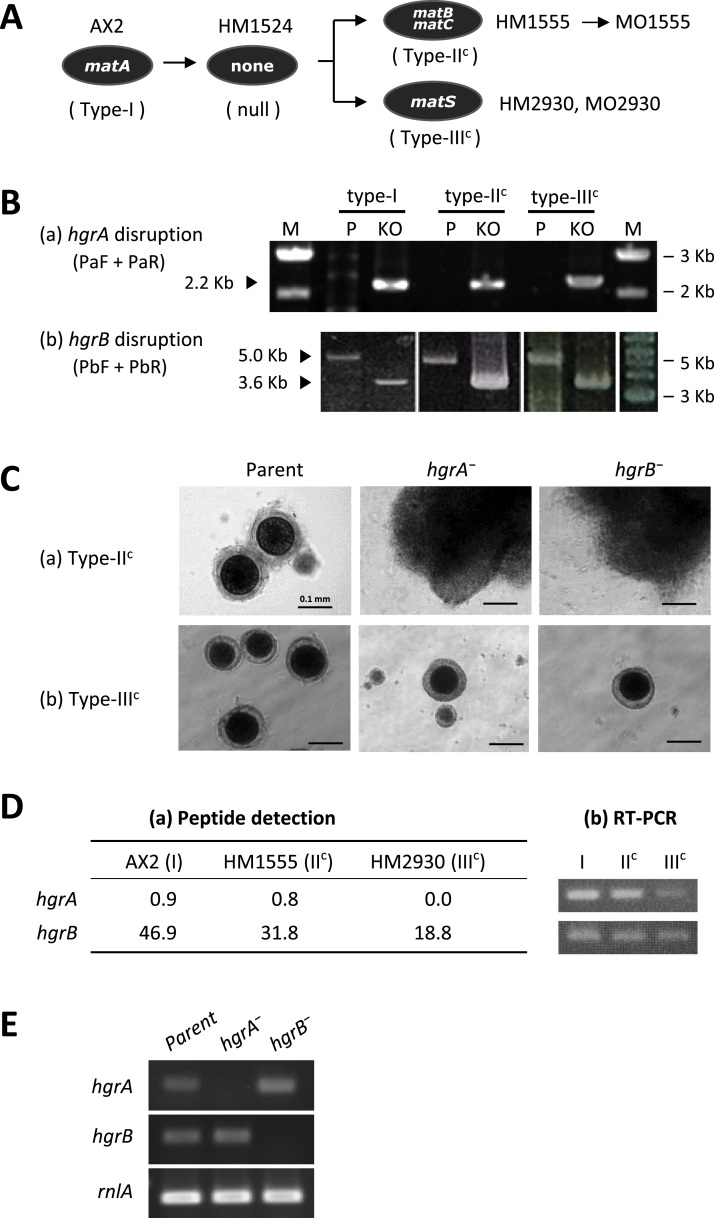
Mating-type dependence of HgrA and HgrB. A: The strain lineages are shown. The *mat* locus genes are indicated within the black ovals for the strains labeled above or to the right of the ovals. Expected mating types are shown in parentheses. We removed the blaS-resistance gene (*bsR*) from HM1555 by the activation of Cre recombinase so that the same knockout constructs could be used as those for KAX3. To avoid spontaneous loss of the extrachromosomal vector harboring the *matS* and *neoR* genes in HM2930, we generated MO2930, using the integration-type vector pTMV18 with the relevant genes. B: Disruptions of *hgrA* (a) and *hgrB* (b) were confirmed by PCR. The primer sets indicated in parentheses are shown in Fig. 2A. The parents (P) for type-I, -II, and -III were KAX3, MO1555, and MO2930, respectively. Type-I samples are included for comparison. C: Growth-phase cells (IC-cells) of each strain were mixed with AX2 cells in a *K. aerogenes* suspension in BSS and cultured under darkness at 22 °C. Photographs were taken after 4 days. D: (a) Data of HgrA and HgrB hits were extracted from the proteome analysis results of the congenic strain set (Table S4). Proteome analysis results of HgrA and HgrB are shown for the congenic strain set. Peptide detection number is normalized for 1×10^5^ total peptides in each sample. (b) Sequences for *hgrA* and *hgrB* were amplified using gamete cDNA as template and primer sets shown by dark red arrowheads in Fig. 2. The sizes of *hgrA* and *hgrB* bands are approximately 300 bp and 270 bp, respectively. E: Expression of *hgrA* (a) and *hgrB* (b) were examined in the type-III^c^ disruptants. RT-PCR was carried out using the gamete cDNA of strains shown above the electrophoretogram and primer sets shown by light red arrowheads in Fig. 2.

Given that HgrA and HgrB were indispensable for mating of type-I strain, the next question was whether their functions were restricted to a single sex, as is observed for plant HAP2-GCS1. We generated the mating-type “congenic” strains derived from AX2 (type-I) (Fig. 3A) and used them instead of the standard tester strains owing to the low efficiency of transformation and homologous recombination in non-axenic strains. We confirmed gene disruption by PCR following the procedures described for the generation of type-I disruptants (Fig. 3B). As shown in Fig. 3Ca, both *hgrA* and *hgrB* knockouts in type-II^c^ completely lacked mating ability with type-I. Their macrocyst formation with type-III was also inhibited (data not shown). However, the disruption of either gene in type-III^c^ did not hamper macrocyst formation with type-I (Fig. 3Cb). Mating ability with type-II was also unaffected (data not shown). These results clearly demonstrate that the functions of both HgrA and HgrB are mating-type specific; they are indispensable for mating of type-I and type-II^c^ but not of type-III^c^.

Since necessity of HgrA and HgrB for mating of type-II^c^ was rather unexpected from their trace expression in V12, a wild type tester strain of type-II (Fig. 1C), we examined their expression in the congenic strain set. As shown in Fig. 3Da, HgrA detection in the gamete membranes was very low and sex-specificity was uncertain as in the tester strain set. On the other hand, HgrB detection was different from that of wild type tester strains; it was highest in type-I, lowest in type-III^c^, and at the average level in type-II^c^. Relative expression of genes as revealed by RT-PCR was nearly in parallel with that of proteins in the gamete membranes (Fig. 3Db). Thus, type-II wild type and congenic strains appeared unequal in the expression of HAP2-GCS1, albeit the mating behavior was completely the same.

There could be a possibility that the remaining gene complemented the counterpart loss in case of type-III^c^ disruptants. We observed some increase in *hgrA* expression in the *hgrB*-disruptant compared to the parent (MO2930) (Fig. 3Ea) but *hgrB* expression in the *hgrA*-disruptant was indistinguishable from the parent (Fig. 3Ea), suggesting that the possibility of complementation is less likely.

## 4 Discussion

Despite extensive diversification of the sexual reproduction system, the essential step is conserved; shuffling of genetic materials following membrane fusion between two separate cells. Therefore, analyses of conjugation mechanisms in simple isogamous organisms can well aid the understanding of those in complex systems. Even apparently specific traits of the former give insights into evolutionary aspects of fertilization. Here we reported both conserved and unique features of gamete interactions in the cellular slime mold. The *D. discoideum* ortholog of HAP2-GCS1, HgrA, plays a critical role for zygote formation mating-type dependently. This was the first demonstration for involvement of HAP2-GCS1 in amoebozoa mating, strengthening the broad conservation of its function across phyla with deep root. Another finding of ours was that the more distant homolog, HgrB, was also indispensable for gamete fusion. Homology of this protein is limited to the HAP2-GCS1 motif region, and overall sequence is unique to dictyostelid lineage, supporting the essential function of this motif (Mori et al., 2010, Ebchuqin et al., 2014). Finally, the multiple mating types in *D. discoideum* fell into 2 groups, HAP2-GCS1 requiring (type-I and -II) and not requiring (type-III) for gamete fusion, analogous to male *vs*. female in plants or plus *vs,* minus in algae.

**Fig. 4:**
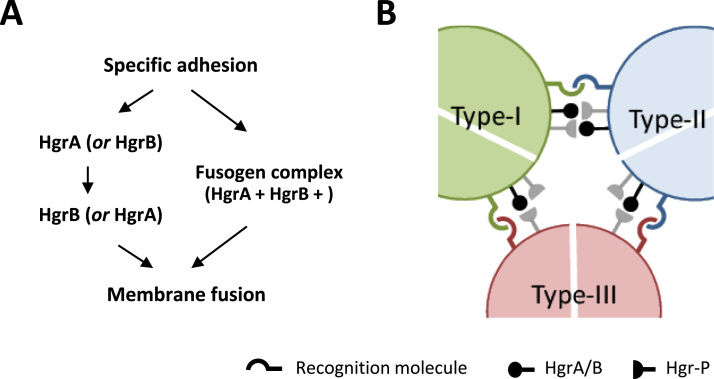
Hypothetical schemes for mating-type specific gamete fusion in *D. discoideum*. A: Two possibilities for involvement of HgrA and HgrB are shown. They may function sequentially (left) or simultaneously in the multi-component fusogen complex (right). B: A model for mating-type specific gamete fusion is illustrated. Type-I, -II, and -III gametes are shown in green, blue, and pink, respectively. Type-specific adhesion allows fusogen interactions and also inhibits self-mating of type-I and type-II gametes.

Reason for necessity of two related proteins in *D. discoideum* mating is currently elusive; they may function either sequentially or in multi-component fusion machinery (Fig. 4A). If the latter is the case, differential peptide detection between HgrA and HgrB suggests the possibility that the fusogen complex is only assembled locally at the fusion sites triggered by the interaction with a complementary gamete, like the HAP2-GCS1 transport in plant (Sprunck et al., 2012) and algae (Kawai-Toyooka et al., 2014). It may be worth mentioning here that MatD and MatT encoded within the mating-type loci of type-II and -III, respectively, also contain sequences weakly homologous to the HAP2-GCS1 motif with conserved cysteine residues. They were not responsible for mating-type determination but did enhance macrocyst formation (Bloomfield et al., 2010), and could be counterparts of HgrB in type-II and -III. Since functional discrimination of HgrA and HgrB is not feasible at the present stage, the term HgrA/B will be used to indicate either of them or their complex. Localization analysis of these proteins, which has not been successful to date, will be one of the most important issues to clarify their functional relationships.

We were able to determine clearly whether or not HAP2-GCS1 homologs function in a sex-specific manner in *D. discoideum*, but a crucial question remains on the mechanism of mating-type specific fusion. Assuming that membrane fusion is mediated by the interaction between HAP2-GCS1 and its partner protein on the complementary gametes as was suggested in plants and algae (reviewed by Mori et al. (2015)), we expect type-III gametes to possess the partner molecule(s) for HgrA/B (tentatively called Hgr-P). Similarly, type-I and -II gametes themselves should retain Hgr-P in addition to HgrA/B for mutual fusion (Fig. 4B), implying the possibility of redundant bidirectional molecular interactions at their interface. The situation coincides with the argument on the conjugation mechanism in *Tetrahymena*, which exhibits 7 mating types, that pore formation leading to membrane fusion is driven on both sides of the nuclear exchange junction (Cole et al., 2014). On the other hand, there is dissimilarity between *Tetrahymena* and *Dictyostelium* in the effect of HAP2-GCS1 deletion; it was dose-dependent in *Tetrahymena* and had to be deleted in both of mating pairs for membrane fusion to be blocked, whereas fusion was completely inhibited by single disruption of HgrA/B in *Dictyostelium*. A possible explanation is that one directional interaction is insufficient to mediate membrane fusion in *D. discoideum* which has no specialized conjugation structures. Type-III may have some modification in the fusion machinery to solve this problem.

Although GCS1 and HAP2 were named after their specificity to generative haploid cells (Mori et al., 2006, von Besser et al., 2006), expression specificity of HAP2-GCS1 homologs in *D. discoideum* was less remarkable. We noticed that part of the *hgrA* sequence had been cloned in a gamete-enriched cDNA library as FC-IC0522, but this clone was not analyzed further because the up-regulation upon sexual maturation was not high enough (FC/IC=2.0) (Muramoto et al., 2003), coinciding the present results. The lack of strong specificity to gamete-phase FC-cells may be related to the nature of *D. discoideum* gametes: First of all, *D. discoideum* cells are haploid throughout the life cycle except for a limited period from zygote formation to dormancy as macrocysts. Next, sexually mature cells do continue proliferation while keeping fusion ability under dark and humid conditions. Muramoto et al., 2003, Muramoto et al., 2005 performed functional analysis of 24 genes highly specific to gametes (5–430 fold increase) but none of them were found essential for mating with type-II cells, even though some were also sex-specific, like *gmsA* (gamete and mating-type specific gene A). Thus, gamete-phase cells in *D. discoideum* seem less specialized than other organisms.

The principal reason for our use of the mating-type congenic strains was to circumvent the low efficiency of transformation in non-axenic strains. In addition to this, their common genetic background, except for the mating-type locus, should be beneficial for analytical studies like comparative proteomics as described below. On the other hand, it should be pointed that we observed differences between tester and congenic strain sets in protein and gene expressions; expression of HgrB was more repressed in V12 (type-II) than in type-II^c^. In consideration that mating-type locus genes should eventually control genes responsible for specific mating behavior of each sex, a logical explanation for the difference would be that the downstream genes or regulatory elements have accumulated changes in the wild strains, while keeping the mating-type intact. Generation of gene knockouts in tester strains, especially in V12, will clarify some of the questions remain unsolved.

It was shown that HAP2-GCS1 acts for membrane fusion event after sex-specific cell adhesion in *Chlamydomonas* (Liu et al., 2008) and *Tetrahymena* (Cole et al., 2014). Based on these observations, it is likely that other surface components play roles for self-non-self discrimination during *D. discoideum* mating, which could explain the situation that the gametes having both HgrA/B and Hgr-P do not self-mate. Identification and characterization of the hypothetical recognition molecules and Hgr-P are next key issues to be challenged and will clarify questions and hypotheses mentioned above. Expecting that expression of those proteins are under the control of *mat* locus genes, we are performing comparative proteomic analyses of the gamete membranes from *mat* locus congenic strains (Table S4, Fig. S3). Thus far we detected multiple mating-type-specific proteins. About half of them were uncharacterized protein and a high proportion of the rest were involved in intercellular communication (Table S5), both will be good candidates for next analyses.

## 5 Conclusions

Heterothallic strains of *D. discoideum* possess two HAP2-GCS1 homologs. While 2 out of 3 mating-types require both of them for sexual cell fusion, the third one does not, suggesting male-female differentiation in this amoebozoa.

## Figures and Tables

**Table 1:** *Dictyostelium discoideum* strains used in this study.

Strain		Mating type^a^	Parent b	Axenic growth	Drug resistance	Macrocyst formation with			Source
Type I	Type II	Type III
Wild type									
	KAX3	I	NC4	+	None	−	+	+	
	AX2	I	NC4	+	None	−	+	+	
	V12	II	(WI)	−	None	+	−	+	
	WS2162	III	(WI)	−	None	+	+	−	
							
*mat* locus mutants									
	HM1524	null	AX2	+	neoR	−	−	−	Bloomfield et al. (2010)
	HM1555	II^c^	HM1524	+	bsR	+	−	+	Bloomfield et al. (2010)
	HM2930	III^c^	HM1524	+	neoR	+	+	−	Bloomfield et al. (2010)
	MO1555	II^c^	HM1555	+	None	+	−	+	This study
	MO2930	III^c^	HM1524	+	neoR	+	+	−	This study

a Based on *mat* locus gene types. II^c^ and III^c^ indicate *mat*-locus congenic.

b WI: wild isolate.
