# Supplementary material for: Two HAP2-GCS1 homologs responsible for gamete interactions in the cellular slime mold with multiple mating types: Implication for common mechanisms of sexual reproduction shared by plants and protozoa and for male-female differentiation
Source: Dev Biol. 2016 Jul 1;415(1):6–13. doi: 10.1016/j.ydbio.2016.05.018 (PMC4910948; doi:10.1016/j.ydbio.2016.05.018)
Supplement: Supplementary file 1 — Supplementary material [file mmc1.zip › SM1.pdf]

A

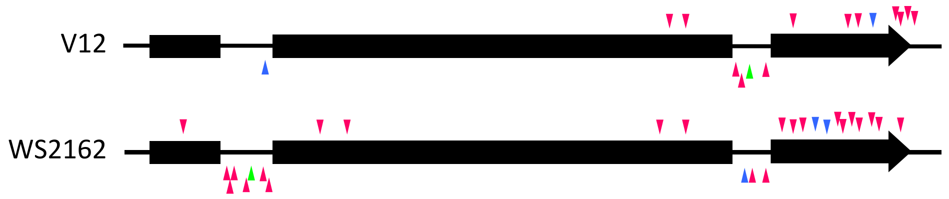

B

Position	KAX3	V12	WS2162
145	I	*	T
479	N	*	I
535	C	*	G
548	T	A	A
553	A	*	T
616-643	(N-stretch variation shown in **C**)		
678	F	L	*

C

```
K: 614 YNPNNPNNNPNNNPNNNNNNNNFRNRNNNN--------------------------------FR 645
V: 614 YNPTNPTNNPNNNPNNNNNNNNFRNRNNNNNNNNNNNNNNNNNNNNNNNNNNNNNNNNNNNNNFR 677
W: 614 YN---PINNPNNNPNNNPNSNNFRNRNNNNNNNNNNNNNNNNNNNNN---------------FR 659
       **   * ********** *.**********                                **
```

Figure S1. Sequence variation of *hgrA* among wild type strains. Genome sequences of *hgrA* in V12 (type-II) and WS2162 (type-III) were determined and compared with KAX3 sequence. **A**: Nucleotide variations are plotted on the schematic illustration of KAX3 genome. Pink, blue and green triangles represent single nucleotide substitution, insertion, and deletion, respectively. **B**: Amino acid variations are listed. An asterisk indicates same with KAX3. No variations were observed in the signal peptide (1-27), HAP2-GCS1 motif (283-331), and transmembrane regions (555-577). **C**: Regions containing N-stretch variation are aligned. K, V, and W stand for KAX3, V12, and WS2162, respectively. Green letters indicate neighboring conserved regions.

A

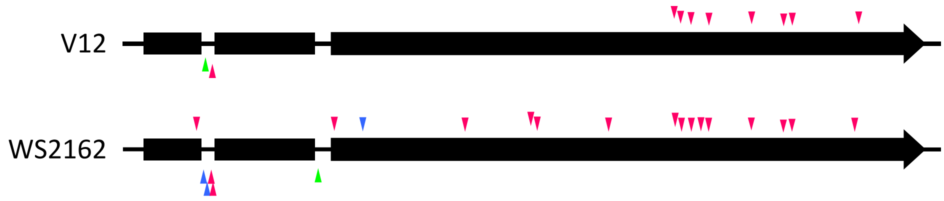

B

Position	KAX3	V12	WS2162
83	S	*	L
449-451	DDD	*	DDDD
820	I	*	K
882	Y	*	N
1249	I	N	N
1319	W	C	C
1419	Q	H	H
1671	L	F	*

Figure S2. Sequence variation of *hgrB* among wild type strains. Genome sequences of *hgrA* in V12 (type-II) and WS2162 (type-III) were determined and compared with KAX3 sequence. **A**: Nucleotide variations are plotted on theKAX3 genome. Pink, blue and green triangles represent single nucleotide substitution, insertion and deletion, respectively. **B**: Amino acid variations are listed. No variations were observed in the signal peptide (1-25), HAP2-GCS1 motif (496-556), and transmembrane regions (1586-1608).

A

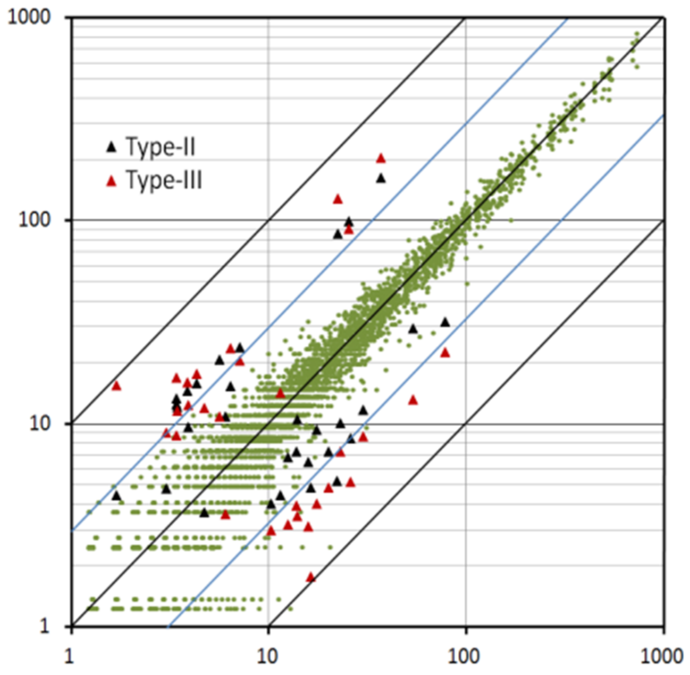

B

Strain	Mating type	Number of specific proteins		
		Enhanced	Repressed	Total
AX2	I	16	23	39
HM1555	II[c]	0	1	1
HM2930	III[c]	5	2	7
	Total	21	26	47

Figure S3. Mating-type dependence of gamete membrane proteins. A: Peptide hits of proteins selected as type-specific (See below.) were overlaid on the background variation for AX2 (green dots). Blue and black diagonal lines indicate 3-fold and 10-fold differences, respectively. Black and red triangles indicate the average detection for type-II, and type-III. **B**: Numbers of specific proteins are summarized for each strain.

Specificity criteria: We set the lower limit for the significant detection of proteins to 3 peptides per 100,000 total hits. Altogether, 1,921 proteins met this criterion in at least one strain. Variation in peptide detection among strains was mostly within 3-fold, except for the low-hit regions (<10), corresponding to background variation observed for repeated measurements within each strain. Proteins with significant peptide hits in one strain but not in the other strains were also regarded as type-specific.

Table S1. Primer sequences used in this study

#	Primer	Alias[a]	Sequence (5' to 3')
For determination of genomic sequences[b]			
1	hgrA-fragment1-F		CTTTCTCAGTAATCGAGTTAGCCAATTC
2	hgrA-fragment1-R		GACTGTCTCTAATTAATTTACCATTGGC
3	hgrA-fragment2-F		GGAAAATTCAGAAACATTTAATTTTCAAGTTAGTG
4	hgrA-fragment2-R		GAGTTATCACCAGATTGAGCACC
5	hgrA-fragment3-F		CAATCAATGCAGACTATACAATGACAATC
6	hgrA-fragment3-R	PcF	GAAAGTTCGAGAAATTATTAATCGATAATATTGAAG
7	hgrB-fragment1-F		CAGTAATTGAGTTCTCCAAATCAAAAAACC
8	hgrB-fragment1-R		GTTAATTGAACTTCATAGGGTGGTG
9	hgrB-fragment2-F		CATTAAAAGTAGAATTATGTTCAACTATAAGTGATTC
10	hgrB-fragment2-R		GTACCTGCTATACCAACCCAAG
11	hgrB-fragment3-F		GATACCAAATTGGATAAATGTTGGTGG
12	hgrB-fragment3-R		GGAATTGTAATTGTATATGAATCAGCCC
13	hgrB-fragment4-F		CCATCAATGGATAGTAATCCATTACC
14	hgrB-fragment4-R		CTTGAGGTGGAGTTGTATAAATTGG
15	hgrB-fragment5-F		CAATTTTTAGTTTTACCAAAATTATGTTGGTCAAC
16	hgrB-fragment5-R		CAATTATTATTGGGGTTATAAAATTGTGTATAAAAGAC
For preparation of KO constructs[c]			
17	hgrA-arm1-F		CAAAAATGACCAGGATTTCC
18	hgrA-arm1-R		GTAATCATGGTCATAGCTGTTTCCTGCAGGGCATCTATTGCACCTGGAC
19	hgrA-arm2-F		CACTGGCCGTCGTTTTACAACGTCGACAATGGATATTCGCACCCAGG
20	hgrA-arm2-R		CACATGCGGTTTGTCACATC
21	hgrB-arm1-F	PbF	GTTGAGCCACCATCAGGTAC
22	hgrB-arm1-R		GTAATCATGGTCATAGCTGTTTCCTGCAGAGGTGCATTTTGAGGATCAG
23	hgrB-arm2-F		CACTGGCCGTCGTTTTACAACGTCGACGTGGATGGGATCCTTTATTG
24	hgrB-arm2-R	PbR	ATCCAAGTATCCCTTGGGTC
25	ddcB-F	PaF	AACTTCAGCACCAAGSCCAG
26	bsR-R	PaR	ACAATCTGGTGCATAGTCTG
For preparation of overexpression constructs[d]			
27	hgrA-ORF-F		TTGGGGATCCATCAAAAAATTGATCTTTTTTTC
28	hgrA-ORF-R	PcR	TTGGCTCGAGTTAGTTTTTTAAAATGAAACC
29	hgrA-ORF-1-F		GGTTGGATCCAAAAAAAATGATCAAAAAATTGATCTTTTTTTC
30	hgrA-ORF-1-R		GGTTTCTAGAGTTTTTTAAAATGAAACCATTTTTTGG
For RT-PCR[e]			
31	hgrA-1F	(dark red)	TGGTGTGGAATCATTTCGTCAAGCTC
32	hgrA-1R	(dark red)	CAGTTTCTTTTGGTTGATAAGCTACATTTTGTAC
33	hgrB-1F	(dark red)	CATATATTGTTGAGCCACCATCAGGTAC
34	hgrB-1R	(dark red)	GTTAACCAAATTCTATTACCAACTCCCTTTG
35	hgrA-2F	(light red)	ATGAAAGATTTAACCCATTATAATCC
36	hgrA-2R	(light red)	GCCACCTGGTTCCAATTTTAG
37	hgrB-2F	(light red)	AGGCAAAGAGCCATTGATTG
38	hgrB-2R	(light red)	CCAATATCAATTGTAAAATCACCAG

[a] Short name or symbol used in Fig.2A.
[b] Genomic sequences of hgrA and hgrB were cloned in overlapping 3 and 5 fragments, respectively.
[c] arm1 and arm2 correspond to left and right arms, respectively.
[d] 2nd set (29-30) is for fusion to FP (stop codon was removed)
[e] 4th and 5th sets (35-38) is to amplify the region lost in KO strains

Table S2. Accessions for the sequences described in the text.

Species	Strain	Accession
hgrA CDS		
Dictyostelium discoideum	KAX3	LC075764
Dictyostelium discoideum	V12	LC075765
Dictyostelium discoideum	WS2162	LC075766
hgrB CDS		
Dictyostelium discoideum	KAX3	LC075767
Dictyostelium discoideum	V12	LC075768
Dictyostelium discoideum	WS2162	LC075769
Hap2/GCS-1 ortholog		
Dictyostelium discoideum	AX4	XP_643321.1
Dictyostelium purpureum	*	XP_003291595.1
Dictyostelium fasciculatum	*	XP_004359139.1
Polysphondylium pallidum	PN500	EFA79298.1
Physarum polycephalum	*	BAE71144.1
Acanthamoeba castellanii	Neff	XP_004341525.1
Acytostelium subglobosum	LB1	XP_012759224.1
Tetrahymena thermophila	SB210	XP_001030543.2
Trypanosoma grayi	*	XP_009307054.1
Lilium longiflorum	*	BAE71142.1
Arabidopsis thaliana	*	AAY51998.1
Chlamydomonas reinhardtii	*	XP_001695893.1
Trypanosoma brucei gambiense	DAL972	XP_011778472.1
HgrB		
Dictyostelium discoideum	AX4	XP_640501.1
Dictyostelium purpureum	*	XP_003288227.1
Polysphondylium pallidum	PN500	EFA81296.1
Dictyostelium fasciculatum	*	XP_004358104.1
Acytostelium subglobosum	LB1	XP_012756004.1
FC-IC0522 (part of *hgrA*)		
Dictyostelium discoideum	KAX3	AU271668, AU271669

Table S5. Mating-type dependent expression of gamete membrane proteins

Gene_name[a]	Peptides per 100000			Chr[b]	Gene product / Description[c]
	Type-I	Type-II[c]	Type-III[c]		
Enhanced in type-I					
**DDB_G0270774**	35.8	8.2	9.0	1	*
**DDB_G0272901**	88.7	21.2	3.6	2d	EGF-like domain-containing protein
**comF**	18.8	1.6	0.0	2d	REMI mutant fails to aggregate; contains a predicted signal peptide
**DDB_G0273149**	18.8	3.3	1.8	2d	putative importin subunit alpha A
**DDB_G0273483**	64.8	19.6	7.2	2d	*
spkA	32.4	1.6	3.6	2d	member of the TKL (tyrosine kinase-like) group and the ARK (ankyrin repeat-containing kinase) family
psiL	25.6	3.3	7.2	2	PA14 (anthrax protection antigen) domain-containing protein
**DDB_G0281313**	18.8	4.9	0.0	3	*
**DDB_G0281207**	76.8	3.3	0.0	3	EGF-like domain-containing protein
DDB_G0284169	18.8	1.6	1.8	4	transmembrane protein containing 3 predicted transmembrane domains and an additional putative signal sequence
DDB_G0285045	25.6	8.2	0.0	4	EGF-like domain-containing protein
DDB_G0285255	29.0	1.6	1.8	4	*
DDB_G0286669	20.5	1.6	1.8	4	acyl-CoA oxidase
DDB_G0286867	27.3	6.5	5.4	4	EGF-like domain-containing protein
**accA**	30.7	4.9	5.4	5	acetyl-CoA carboxylase
DDB_G0287873	23.9	4.9	1.8	5	*
Enhanced in type-III					
DDB_G0270644	5.1	8.2	41.2	1	*
**DDB_G0272546**	6.8	17.9	62.7	2	*
ddx18	3.4	1.6	25.1	3	putative RNA helicase
dnmA	5.1	6.5	23.3	5	DNA (cytosine-5-)-methyltransferase
DDB_G0293444	8.5	6.5	26.9	6	*
Repressed in type-I					
H3a	13.6	50.5	71.7	1	component of the H2A-H2B-H3-H4 histone octamer
**DDB_G0268580_RTE**	10.2	31.0	64.5	1	partial ORF2 of tRNA-specific non-long terminal repeat retrotransposon TRE3-C
DDB_G0268848	10.2	71.7	77.0	1	putative ATP binding protein
DDB_G0269460	6.8	27.7	34.0	1	*
**DDB_G0267648**	13.6	48.9	46.6	1	*
DDB_G0276097	88.7	350.5	516.0	2	putative transmembrane protein
**DDB_G0276219**	146.7	657.1	824.1	2	putative transmembrane protein
pyr1-3	100.6	402.7	369.1	2	multifunctional enzyme that carries out the three first enzymatic activities of the de novo pyrimidine biosynthetic pathway
**DDB_G0277109**	10.2	31.0	37.6	2	*
DDB_G0280429	3.4	26.1	28.7	3	*
**coq7**	5.1	19.6	19.7	3	ubiquinone biosynthesis protein
wdr3	6.8	22.8	21.5	3	ortholog of H. sapiens WDR3 and S. cerevisiae DIP2
DDB_G0282851	6.8	26.1	23.3	3	*
DDB_G0286153	3.4	39.1	57.3	4	*
**rpb2**	1.7	19.6	28.7	5	RNA polymerase II core subunit
**DDB_G0290381**	17.1	63.6	71.7	5	*
DDB_G0287275	6.8	24.5	37.6	5	*
DDB_G0292188	5.1	22.8	43.0	6	conserved hypothetical Dictyostelium protein; contains a weak von Willebrand factor, type A domain
abpF	15.4	58.7	64.5	6	actin-binding protein that localizes to the centrosome
DDB_G0292714	3.4	24.5	19.7	6	*
DDB_G0292752	6.8	34.2	26.9	6	*
DDB_G0293138	6.8	24.5	25.1	6	similar to a gene called src1 in budding yeast
DDB_G0291782	10.2	50.5	60.9	6	*
Repressed in type-II					
gpaB	30.7	8.2	30.5	2	G-protein subunit alpha 2
Repressed in type-III					
cupE	64.8	52.2	10.7	NE	calcium up-regulated protein
**rliB**	52.9	27.7	7.2	3	repressed after Legionella pneumophila infection; contains a signal peptide; similar to comF

[a] Only those detected at more than 3 fold increase (Enhanced) or decrease (Repressed) compared to the second are shown. Genes that showed the same specificity in the non-congenic tester strain set are shown in bold face.

[b] Chromosomal locations are shown. 2d indicates the duplicated region of chromosome 2 in AX4 (not duplicated in AX2); NE indicates a region that does not exist in the current version of the genome available from dictyBase.

[c] Based on the dictyBase curation.
